# Simulation of the Time Needed for Long-Term Asphalt Ageing in the Rolling Thin Film Oven Relative to That of the Pressure Ageing Vessel

**DOI:** 10.3390/ma16227081

**Published:** 2023-11-08

**Authors:** Yuri Mello Müller de Oliveira, Poliana Tonieto Cittadella, Luciana Rohde, Liseane Padilha Thives

**Affiliations:** Department of Civil Engineering, Federal University of Santa Catarina, Florianópolis 88040-900, Brazil; yurimello@hotmail.com (Y.M.M.d.O.); poliana.tonieto@posgrad.ufsc.br (P.T.C.); l.rohde@ufsc.br (L.R.)

**Keywords:** RTFO, PAV, equivalent time, DSR, LAS, MSCR

## Abstract

Rheological test standards require asphalt samples, both original and under ageing conditions. The most common laboratory equipment in specifications for short-term and long-term ageing simulation tests are the rolling thin film oven (RTFO) and the pressure ageing vessel (PAV). However, the cost of acquiring PAV and the duration of long-term ageing tests can be a limiting factor. This work aimed to establish the equivalent time of the long-term ageing test in the RTFO that corresponds to the PAV. For this, the Brazilian asphalt PEN 50/70, specified by penetration, was aged at different times (85, 170, 255, and 340 min) in the RTFO at the standard temperature (163 °C). For each time, using a dynamic shear rheometer (DSR), tests such as Multiple Stress Creep Recovery (MSCR) and Linear Amplitude Sweep (LAS) were performed, and the rheological properties (complex modulus (G*) and phase angle (δ)) were measured. The same tests were conducted on the samples aged in the long term and in the PAV. The test parameters obtained from applying different times while using the RTFO were compared with the PAV results, and the equivalent time was settled through linear regression, resulting in 300 min. In order to confirm the equivalent time, samples aged in the RTFO for 300 min were assessed using the same rheological tests, and the parameters were compared to those obtained after PAV ageing. At the equivalent time, the difference between RTFO and the PAV for the rutting parameter (G*/sinδ, 58 °C) was 6%, while for the fatigue parameter (G*.sinδ, 19 °C), the difference was 1.0%. The MSCR non-recoverable creep compliance parameter differences, considering stress levels of 0.1 kPa and 3.2 kPa, were 9.7% and 11.7%, respectively. For the fatigue life obtained in the LAS test at strain levels of 1.25% and 2.5%, the difference between RTFO and PAV, at the equivalent time, was 7.6% and 7.8%, respectively. For the Brazilian unmodified asphalt PEN 50/70 and parameters evaluated in this work, 300 min is the equivalent time that simulates long-term ageing in the RTFO.

## 1. Introduction

Proper asphalt characterization in virgin and ageing conditions is essential to predict and ensure the asphalt mixture’s mechanical behaviour in the field. The asphalt ageing process during asphalt mixture production and construction (short-term) and the pavement service life (long-term) must be evaluated, considering that it negatively influences pavement performance [1,2,3].

In order to give a good indication of the ageing effects, asphalt is conditioned in the laboratory. Thin film oven (TFO) and rolling thin film oven (RTFO) tests are commonly used to simulate short-term ageing, while the pressurized ageing vessel (PAV) is used for long-term ageing in binder specifications [4,5]. The ASTM D2872 standard [6] states that the RTFO test should be conducted at 163 °C for 85 min. The ASTM D6521 standard [7] contains guidelines for the standard PAV test, in which the temperatures range between 90, 100, and 110 °C (depending on the climatic conditions), with a time duration of 20 h under an air pressure of 2.1 MPa.

An alternative to the RTFO and PAV tests is the Universal Simple Aging Test (USAT), developed to simulate asphalt oxidative ageing during warm mixture production and when highly modified asphalts are used. Using the USAT for 50 min at 150 °C to simulate short-term ageing is equivalent to using the RTFO for 85 min at 163 °C, while regarding the long term, 8 h at 100 °C is similar to using the PAV for 20 h at 100 °C [8]. The USAT has no standard specification and is not a common test in developing countries, unlike the TFO, RTFO, and PAV.

Asphalt short-term ageing occurs very fast since, during mixture production, a very thin film of asphalt is exposed to air at high temperatures, leading to changes in the chemical component’s composition that affect the rheological properties. On the other hand, in long-term ageing, the asphalt is exposed to the environment (temperature, ultraviolet rays, rainfall) and traffic during service life [9].

Aged asphalt can become highly brittle, resulting in reduced penetration and ductility, increased stiffness, and the promotion of viscoelastic property changes during service life, which can cause pavement distress [10,11,12]. Also, after ageing, asphalt rheological parameters such as complex modulus (G*) and creep recovery rate increase, while the non-recoverable creep compliance and phase angle decrease (δ) [13,14].

From the standard test procedures, it is expected that the RTFO can simulate two years or less of ageing, and the PAV can simulate five to ten years of ageing in the field, although the ageing depends on many factors, such as the asphalt mixture’s characteristics, traffic, and the local climate [15]. Some studies to verify the accuracy between the actual ageing time (field) and the corresponding time in the laboratory tests (RTFO and PAV) have been conducted.

Mallick and Brown [16] aged asphalts in the laboratory and compared them with samples taken from the field in the first three years of the pavement’s life. The results showed that the asphalt underwent more pronounced ageing during the machining process than simulated in the RTFO. Lu et al. [17] collected field core samples from roads in Sweden and compared them to asphalt samples after the use of the PAV. It was concluded that, after 10 to 30 years, the extracted asphalts presented a relatively low degree of age-hardening related to those aged at the laboratory. The field asphalt mixtures’ low void content could contribute to oxidation reduction. Considering long-term ageing, the field’s oxidation mechanisms may differ from the laboratory ageing tests.

Behera et al. [18] extracted and recovered the aged asphalt from field asphalt mixture cores. The same virgin asphalt type was subjected to short-term ageing in the RTFO. Six asphalts (modified and unmodified) were tested for their rheological properties, and the results were compared. They stated that the actual short-term ageing (field) was less than the simulated ageing in the laboratory (RTFO).

Physical hardening is evidenced by the increase in viscosity and stiffness in aged asphalts. However, the oxidative processes promoted by thermal reactions between atmospheric oxygen and asphalt components also influence ageing. In this way, asphalt ageing evaluations should include ultraviolet (UV) radiation simulation since, despite effects mainly affecting the upper surface layer, they differ from those found in the conventional thermo-oxidative tests [1]. Petersen [19] added that oxidative ageing includes photo-oxidation reactions affecting the upper part of the surface layer and thus the asphalt in this specific position. Also, UV radiation penetration is limited to a few micrometres, while the oxygen penetration is in millimetres order.

Abdullah et al. [20] added that the short-term ageing protocol (ASTM D2872 [6]) is not adequate for the simulation of asphalt’s short-term ageing in tropical regions since high daily temperatures, humidity, and ultraviolet radiation significantly affect the physical and rheological properties of the asphalt. Smith et al. [21] found that, through using samples extracted from a pavement surface layer aged 6.5 years, a PAV test protocol of 20 h simulated two years of ageing. For asphalts extracted from the top, in a depth of 1.3 cm, 21 h in the PAV represented two years of ageing in the field, and 45 h in the PAV represented four years of ageing in the field. Other researchers corroborated this and asserted that a single PAV cycle of 20 h does not always represent the asphalt service life and that PAV ageing cycles should be at least 40 or 60 h [22,23].

Although the RTFO and PAV were not designed for porous mixtures, Jing et al. [24] conducted ageing tests for comparison. In their study, asphalts aged in the RTFO after the PAV were equivalent to those extracted from porous surface layers with three years of in-service in the Netherlands. They concluded that field ageing was far more severe than that simulated in the laboratory tests using the RTFO and PAV.

Some tests of the North American specification (Superior Performing Asphalt Pavements—SUPERPAVE) [25] require long-term aged samples (RTFO and PAV). Examples are the rheological tests in the DSR to measure the fatigue parameter and the low-temperature parameters for Performance Grade (PG) establishment using the Bending Beam Rheometer (BBR) and Direct Tension Tester (DTT). The Linear Amplitude Sweep (LAS) test also needs asphalt samples in long-term ageing conditions.

Brazilian asphalts are classified by penetration grade, and since 2009, a few SUPERPAVE tests have been included in the specification [26], such as apparent viscosity measured in a rotational viscometer and short-term ageing measured in the RTFO. The Brazilian specifications still do not include long-term ageing evaluations or rheological tests. On the other hand, comprehensive and advanced analyses of asphalt’s behaviour is necessary, as these are already being performed in developed countries. For this, one of the basic needs is that the laboratories must have the equipment to simulate the ageing conditions.

However, the laboratories often do not have all the needed equipment to carry out the tests. Unfortunately, this scenario is a reality in many developing countries’ laboratories due to the high cost of some pieces of equipment. In this way, simulating long-term ageing using the RTFO could be a viable alternative. In this way, previous studies have investigated the test time that the RTFO could represent the PAV.

In India, to simulate the RTFO and PAV tests, a low-cost method was developed using a standard laboratory oven. The comparison was achieved through SUPERPAVE rheological parameter evaluation. This study showed that for the fatigue parameter, 3 to 4 days of oven ageing (85 °C) could produce long-term ageing similar to the PAV for unmodified asphalts, while 4 to 5 days was required for the modified ones. For the SUPERPAVE rutting parameter, ageing via placing samples in the oven for 180 min and 210 min (163 °C) was similar to using the RTFO for unmodified asphalts (viscosity-graded (VG)—VG30 and VG40, respectively), and an equivalent time of 270 min was found to be optimal for the modified ones [18].

Migliori and Corté [27] used French asphalt (PEN 35/50) to determine whether the PAV test would provide similar results to the RTFO results. The asphalts were subjected to ageing using the PAV, as were similar samples after using the RTFO before the PAV time was increased. For the same conditions, for the studied asphalts, the authors considered an equivalence between the effects of (i) RTFOT and 5 h of PAV ageing at 100 °C and under 2.1 MPa and (ii) RTFOT + 20 h of PAV and 25 h of PAV to be acceptable.

Nagabhushanarao and Vijayakumar [28] evaluated the long-term ageing time duration similar to using the PAV via cycles of using the RTFO (85 min at 163 °C). Two unmodified asphalts (PG 70-XX) from different sources and a modified styrene–butadiene styrene (PG 64-XX) were tested. The comparison was facilitated through obtaining laboratory test results for apparent viscosity, rheological parameters (rutting and fatigue parameters), and Fourier-transform infrared spectroscopy (FTIR). For the SUPERPAVE fatigue parameter (G*.sinδ), the rheological results indicated that, using the RTFO for a duration of 320 min for the unmodified asphalts and 360 min for modified PG 64-XX asphalt could be an alternative to using the PAV. For the SUPERPAVE rutting parameter (G*/sinδ), the duration in the RTFO resulted in 256 ± 6 min for both PG 70-XX asphalts and 269 min for the modified asphalts. Based on the chemical analysis results, for unmodified asphalt PG 70-XX, an RTFO duration of 255 min was equivalent to PAV.

Pasetto et al. [29] asserted that asphalts subjected to the RTFO for an extended amount of time (325 min) have similar characteristics (penetration and softening point) and properties (stiffness and fatigue resistance) compared with to PAV residues.

Studies to simulate short-term ageing in the PAV based on conventional test (penetration, softening point, and apparent viscosity) results for unmodified asphalts (PEN 80/100 e PEN 60/70) have also been performed. Saleh [30] showed that five hours in the PAV was equivalent to short-term ageing in the RTFO. Through macroscopic rheological and microchemical evaluations, Tian et al. [5] concluded that, for modified asphalts, RTFO ageing was equivalent to five hours of using the PAV.

In Hong Kong (subtropical climate), Qian et al. [31] compared asphalts (PEN 60/70) extracted from the cores of pavement surface layers (five to four years in-service) with asphalts aged at the laboratory (via the RTFO and PAV). The asphalts (aged in the laboratory and extracted from the field) were subjected to rheological tests using a dynamic shear rheometer and Fourier-transform infrared spectrometer. The comparison showed that laboratory ageing methods underestimated field ageing.

In the United States of America (Illinois), asphalts extracted from field cores aged 8 to 31 years were tested for their rheological and chemical characteristics. The authors of the study concluded that the long-term laboratory ageing test (PAV, 40 h) represented 8 to 12 years of field ageing for Illinois pavements [32].

The referenced studies corroborated that the asphalt ageing process is complex, and its understanding is essential to predict material behaviour and pavement performance.

There is still no consensus among researchers regarding representing the simulation of the asphalt ageing process that occurs during asphalt mixture production (short-term) and in service in the field (long-term) through laboratory ageing tests. It is important to highlight that the laboratory tests were developed for specification compliance. For instance, the Strategic Highway Research Program (SHRP) adopted the RTFO test due to the large amount of aged asphalt needed for low-temperature tests in the BBR [8]. Despite not always being representative, laboratory evaluation is required, and the results indicate the material’s behaviour over the ageing process stages and assess whether the material fits the relevant specifications.

Due to scarce resources to develop research, developing countries face challenges, and consequently, some tests are not conducted with the required frequency. Because of this limitation, acquiring all the testing equipment needed for asphalt specifications is often impracticable, resulting in inadequate pavement performance in the field.

Despite the available techniques and the different tests previously studied for reproducing long-term asphalt ageing in equipment that simulates short-time ageing, the RTFO is vastly widespread and known. Considering that, commonly, in developing countries such as Brazil, laboratories have the RTFO and can perform the test, it was chosen in this study.

This work aims to establish the equivalent time of the long-term ageing test in the RTFO that corresponds to the PAV. The unmodified Brazilian asphalt (PEN 50/70) was aged at different times in the standard RTFO test. The rheological parameters were evaluated, and the results were compared to those obtained with the samples aged in the standard PAV test. The results could help to provide an alternative for developing countries to conduct long-term ageing tests in unmodified asphalts using cheaper equipment (RTFO).

## 2. Materials and Methods

### 2.1. Materials

An unmodified asphalt PEN 50/70, classified by penetration grade, was selected because it is the most used in Brazil. The characterization tests (Table 1) indicated that PEN 50/70 fit the Brazilian specifications [26].

### 2.2. Methods

**Figure 1:**
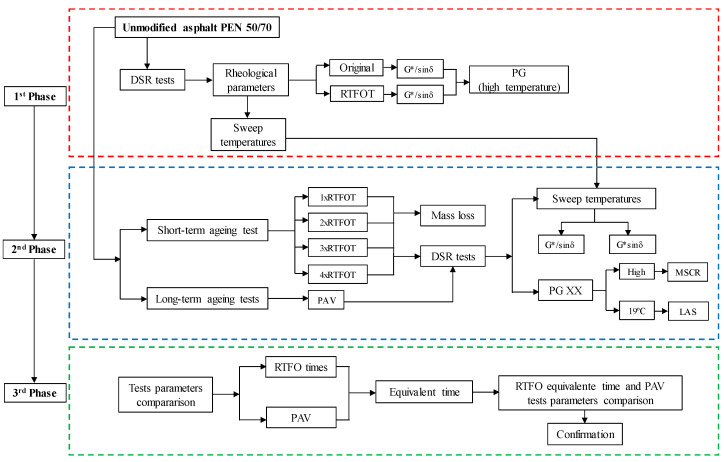
Flowchart of the experimental program.

Figure 1 presents a flowchart showcasing the experimental program used to establish the equivalent time between the RTFO and PAV, which was divided into three main phases.

In the first phase, the asphalt PEN 50/70 (penetration grade) was classified according to the SUPERPAVE specification (Performance Graded (PG) [25]). The rheological parameters (complex shear modulus (G*) and phase angle (δ)) were measured using a Dynamic Shear Rheometer (DSR). The SUPERPAVE rutting (G*/sinδ) and fatigue (G*.sinδ) parameters were evaluated according to the ASTM D7175 [37] standard in a frequency of 10 rad/s at a range of temperatures.

The tests were conducted until temperature failure according to SUPERPAVE requirements [25], i.e., for original asphalt, the rutting parameter (G*/sinδ) must be greater than or equal to 1.0 kPa and, after RTFO, to 2.2 kPa. For the fatigue parameter (G*/sinδ), using samples tested after PAV, the SUPERPAVE [25] limit is 5000 kPa. The RTFO tests followed the ASTM 2872 [6] standard, and the PAV tests followed the ASTM-D6521 standard [7]. From the test results, the high-temperature PG was established. Tests to measure the parameters of lower temperatures were not performed, considering that Brazil’s climate is equatorial and tropical and that thermal cracking is rare in pavements. Because of this, BBR and DTT tests were not included in our analysis.

In Phase 2, PEN 50/70 was subjected to four (times) cumulative cycles of RTFO equivalent to the standardized test (85 min at 163 °C). The methodological process was similar to that assumed by Nagabhushana and Vijayakumar [28]. Table 2 presents the adopted nomenclature of the tested samples and their corresponding ageing times. After the first RTFO time, PEN 50/70 was subject to a long-term ageing standard PAV test (20 h at 100 °C with an air pressure of 2.1 MPa).

A protocol for both ageing tests was established relative to sample collection and storage. The samples were heated (about 100 °C) until adequate fluid consistency for collection. For the RTFO test, 35 g was poured into each RTFO glass container. Mass loss was measured at the end of each cycle, and then the samples were collected to perform the rheological tests conducted the day after sample collection to reduce the storage time. This protocol was followed for each RTFO test time. After the first RTFO test time, 50 g mass samples (RTFO-aged) were subjected to the PAV test. Similarly, the rheological tests started on the day after sample collection.

All samples from the RTFO and PAV tests were subjected to rheological tests using a DSR to measure the SUPERPAVE parameters (G*/sinδ and G.*sinδ) at a range of temperatures (high and intermediate) previously selected in Phase 1. The tests were conducted at 10 rad/s using 8 mm parallel plate geometry and 2 mm gap for G*.sinδ, and for G*/sinδ, the 25 mm parallel plate geometry and 1 mm gap. Also, the Multiple Stress Creep Recovery (MSCR) and Linear Amplitude Sweep (LAS) tests, according to the ASTM D 7405 [38] and DNIT 439 [39] standards, respectively, were conducted.

In the DSR (25 mm parallel plate geometry and 1 mm gap), MSCR tests [38] were performed by applying the samples at a repeated loading of 1 s, followed by a recovery period of 9 s at a stress level of 0.1 kPa. Then, the same test protocol was used for the stress level of 3.2 kPa. Ten cycles were applied for each stress level, and the test parameters obtained were the percent recovery (R) and the non-recoverable creep compliance for each stress level (J_nr_). The test temperature was the high PG for MSCR.

The LAS test was conducted in the DSR (8 mm parallel plate geometry and 2 mm gap), consisting of an oscillatory strain amplitude sweep and a frequency sweep by systematically increasing load amplitudes under cyclic loading. In the first test, the samples were subjected to a frequency sweep to determine a parameter (a) representing the viscoelastic properties of the non-damaged asphalt. After that, the second test was conducted in oscillatory shear, in strain-controlled mode, at a frequency of 10 Hz. The LAS test temperature established by the Brazilian standard is 19 °C [39].

In the LAS test, the strain was increased linearly from 0.1% to 30% throughout 3100 loading cycles for a total test time of 310 s. Peak shear strain and peak shear stress were recorded every ten load cycles (1 s), as well as the phase angle and complex shear modulus. The LAS test results were analysed using the viscoelastic continuum damage (VECD) model. From the LAS test results, the values for the fatigue life of the asphalt (N_f_), as a function of three strain levels (1.25%, 2.5%, and 5.0%), were established.

The establishment of the equivalent time between the RTFO and the PAV was evaluated in the third phase via linear regression from each of the following rheological test results: (i) G*/sinδ; (ii) G*.sinδ; (iii) J_nr_ for both stress levels; (iv) fatigue life cycles (N_f_). The data are shown graphically, and in the graphs, the horizontal axis represents the RTFO ageing time (minutes), and the longitudinal axis represents the respective parameters obtained in each test. For each graph, the angular (a) and linear (b) coefficients were obtained, and the time equivalent was obtained using Equation (1).

T_eq_ = (P_PAV_ − b)/a (1)

where T_eq_ is the equivalent time from the RTFO, P_PAV_ is the test parameter obtained in PAV, a and b are the regression coefficients.

In order to confirm the estimated equivalent time, PEN 50/70 samples were subjected to the RTFO test (test duration equal to the equivalent time). The asphalt adopted nomenclature was PEN_eq_; the rheological tests were performed, and the results were compared to those obtained in the PAV samples. The percentage difference between the RTFO test carried out at the equivalent time and the PAV was calculated through using Equation (2).

D = [(PAV − RTFO_eq_)/aPAV] × 100 (2)

where D is the difference between the RTFO and PAV results (%), PAV is the parameter result obtained in the PAV test, and RTFO_eq_ is the parameter result obtained in the RTFO test with the duration of the equivalent time.

Additionally, the maximum percentage error of the test parameters, when available and predicted in the standards, was evaluated. The maximum percentage error for asphalt aged in the PAV for G*.sinδ is 13.8%, according to the ASTM D7175 [37] standard. For the MSCR test, the ASTM D7405 [38] standard indicates 38.3% for J_nr_ (stress level of 0.1 kPa) and 26.6% (stress level of 3.2 kPa). The maximum limits of the standards were compared with the percentage of errors obtained in the test parameters.

## 3. Results

### 3.1. Performance Grade (PG)

To establish the high-temperature PG, the asphalt PEN 50/70 was subjected to DSR tests [37] at three temperatures (58 °C, 64 °C, and 70 °C), and the results are shown in Table 3. The results (Table 3) showed that for the temperature of 58 °C, the rutting parameter (G*/sinδ) met the SUPERPAVE [25] limits, both for the original condition (greater than 1.0 kPa), which was 2.06 kPa, and after RTFO (greater than 2.2 kPa), which resulted in 3.72 kPa. The SUPERPAVE limits [25] for this parameter were not met for temperatures of 64 ° and 70 ° (Table 3).

Thus, the Brazilian asphalt PEN 50/70 was classified as PG 58-XX. For PG 58-X, the corresponding intermediate temperatures are 19 °C, 22 °C, and 25 °C [25], and these values were selected to conduct the DSR tests in Phase 2.

### 3.2. RTFO Mass Loss

**Figure 2:**
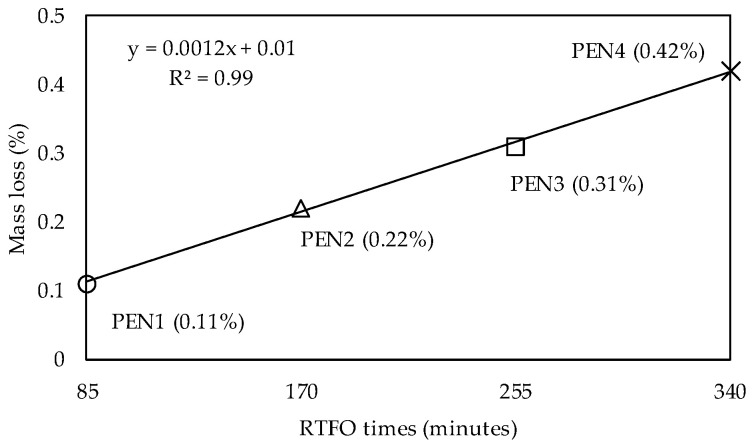
Mass loss at different RTFO times.

Mass loss was measured for each RTFO time (Table 2), and the results shown in Figure 2 correspond to the average of three samples per test. As expected, there was a continuous growth in the percentage of mass loss as the ageing time increased. In the standard test time (85 min—PEN1), the mass loss was 0.11%, while in the four RTFO cycles (340 min—PEN4), it was 0.41%, both below the limit of 1% established in the Brazilian standard [26] and SUPERPAVE specification [25]. Even after 340 min in RTFO, the small mass loss (0.43%) could indicate that the volatile light components evaporated on a constant scale during ageing.

### 3.3. SUPERPAVE Parameters G*/sinδ and G*.sinδ

The complex modulus (G*) and phase angle (δ) results, measured in the DSR to calculate the rutting and fatigue parameters, are presented in Table 4 and Table 5, respectively. For the test temperature of 58 °C, it was noticed that G* increased as the RTFO time increased (Table 4), indicating that the ageing process caused the asphalt to become stiffer. In contrast, a slight variation in the phase angle (δ) was noticed with increasing ageing times (Table 4), suggesting that the isolated analysis of this parameter may not be adequate at high temperatures. The same behaviour was also observed for the other tested temperatures, both for G* and δ.

Additionally, it was noted that there was a pattern regarding the rise in stiffness with the cumulative RTFO cycles. As shown in Table 4, at 58 °C and under the original conditions, G* had a value of 2.06 kPa. Subsequently, relative to the original conditions for the standard RTFO test (85 min), G* increased by 80% (3.72 kPa); at 170 min, it increased by 230%, while at 255 min, it increased by 340% (9.14 kPa), and at 340 min, it increased by 570% (13.88 kPa). For temperatures of 64 °C and 70 °C, in terms of the original conditions, the stiffness increase was, at 85 min, 70% and 60%; at 170 min, the stiffness increase was 200% and 170%, while at 255 min, the stiffness increase was 300% and 260%, and at 340 min, the stiffness increase was 530% and 490%, respectively. The ageing affected the asphalt stiffness, but the percentage growth descended with increasing cumulative RTFO cycles and temperatures.

For intermediate temperatures (Table 5), as the ageing time and temperatures increased, the asphalt stiffness increased, but the phase angle (δ), an indicator of elastic material response, was only slightly reduced.

**Figure 3:**
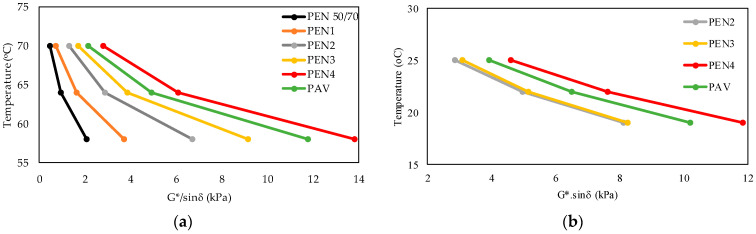
Results of SUPERPAVE parameters after the ageing process. (**a**) Rutting parameter. (**b**) Fatigue parameter.

The rheological SUPERPAVE parameters were measured in the DSR in asphalts subjected to the RFTO times shown in Table 2 and aged in the PAV. The tests were carried out at three high temperatures (50 °C, 64 °C, and 70 °C) and three intermediate temperatures (19 °C, 22 °C, and 25 °C). The comparative results are presented in Figure 3. The rutting parameter (G*/sinδ) was more elevated (Figure 3a) with the increase in temperature and ageing times in the RTFO test. Also, in terms of the equivalent time between the RTFO and PAV tests, the PAV curve was between 255 min (PEN3) and 340 min (PEN4). For the fatigue parameter (G*.sinδ), only PEN2, PEN3, PEN4, and PAV were measured, and Figure 3b presents the results. Similarly, the curves showed that the equivalent time (RTFO and PAV) was between PEN 3 (255 min) and PEN 4 (340 min).

### 3.4. MSCR Test Results

**Figure 4:**
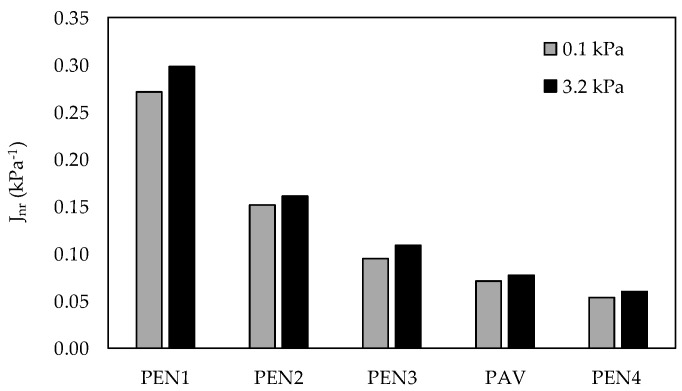
Non-recoverable creep compliance (J_nr_) results for each stress level at 58 °C.

Figure 4 shows the results of the MSCR test for the non-recoverable creep compliance (J_nr_) parameter at 58 °C (high-temperature PG of PEN 50/70), from which it was observed that, for two stress levels, the RTFO ageing times, similar to PAV, were between 255 min (PEN3) and 340 min (PEN4). The increase in RTFO ageing times influenced the material stiffness, which increased, as noted by the J_nr_ reduction. The percent recovery (R) at a stress level of 3.2 kPa indicated the presence of elastic response and stress level dependence; for PEN1, the percent recovery (R) at this stress level, at 58 °C, was 0.51%. As PEN 50/70 is an unmodified asphalt, low performance in an elastic response was expected. Because of this, the recovery parameter was not considered in our analysis. The J_nr_ for both stress levels presented a decedent tendency to increase the ageing level. This behaviour could be explained due to an increase in asphalt stiffness. Since the MSCR test is a test with controlled stress, lower deformation is expected for stiffer materials.

### 3.5. LAS Test Results

**Figure 5:**
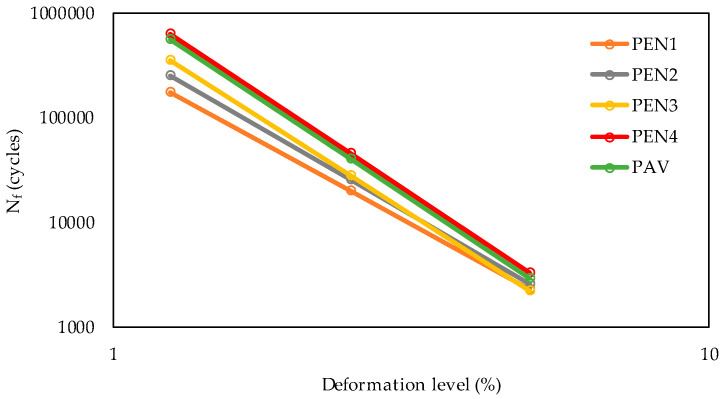
LAS N_f_ parameter results for each deformation level at 19 °C.

For the LAS test, the parameter N_f_ was chosen to evaluate the equivalent time (Figure 5) between the RTFO and PAV tests, which resulted in the range of 255 min (PEN3) and 340 min (PEN4).

In addition, for a deformation level of 5%, the curves (Figure 5) were too close, and for lower deformations (1.25% and 2.5%), the difference was more evident. This can be attributed to the asphalt PEN 50/70 (PG 58-XX) not being resistant enough to support increments of deformations (higher levels) during the test, considering the accumulated damage the samples were subjected to.

### 3.6. Estimation of the Equivalent Time

**Figure 6:**
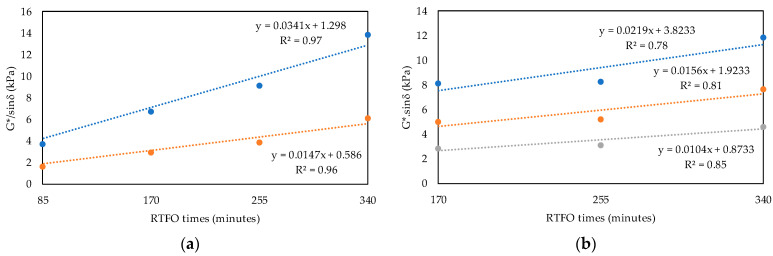
Regression curves for the SUPERPAVE parameters. (**a**) Rutting parameter. (**b**) Fatigue parameter.

To evaluate the equivalent time from the results of each test, linear regression lines were represented in graph form. Figure 6a presents the regression curves of G*/sinδ for the temperatures of 58 °C and 64 °C. As 70 °C exceeds two high PGs (12 °C) for the PEN 50/70 (PG 58-XX), our analysis did not include the rutting parameter for this temperature. Figure 6b shows the regression curves of G*.sinδ for three temperatures (19 °C, 22 °C, and 64 °C).

**Figure 7:**
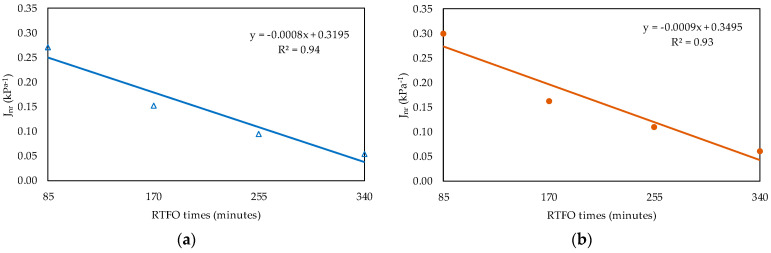
Regression curves for J_nr_. (**a**) Stress level of 0.1 kPa. (**b**) Stress level of 3.2 kPa.

**Figure 8:**
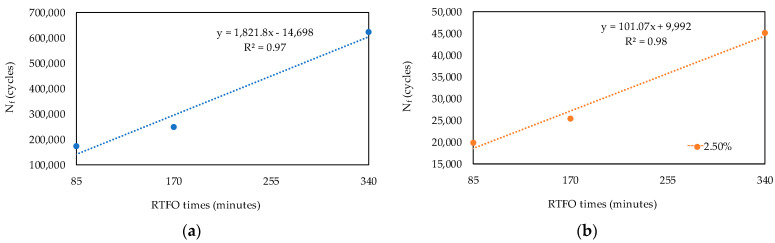
Regression curves for N_f_. (**a**) Deformation level of 1.25%. (**b**) Deformation level of 2.50%.

The regression curve of parameter J_nr_ at 58 °C is shown in Figure 7a for the stress level of 0.1 kPa, and in Figure 7b, that for the stress level of 3.2 kPa can be seen. Figure 8 presents the regression curves of N_f_, the LAS test parameter, for the deformation levels of (a) 1.25% and (b) 2.50%, both measured at 19 °C. The deformation level of 5% was not included in the analysis, considering that PEN 50/70 could not support this deformation level during the test.

From the relationships obtained (Figure 6, Figure 7 and Figure 8), the equivalent time (Equation (1)) for which the RTFO test was similar to the PAV test was estimated, and Table 6 presents the results. In order to calculate the equivalent time, the rheological test results obtained from the regressions were averaged. The mean value was 300 min, with a standard deviation of 8.4 min. Therefore, the obtained equivalent time was 300 min (PEN_eq_).

### 3.7. Equivalent Time Confirmation

The asphalt samples (PEN_eq_) were aged in the RTFO during the equivalent time (300 min), and then, rheological tests were performed. The results were compared to those obtained in long-term ageing using the PAV, and the percentage difference was calculated using Equation (2) (Table 7).

The acceptable range of two test results obtained by the same operator using the same equipment in the same laboratory is provided in some test standards. Based on the acceptance ranges in the relevant standards (ASTM D7175 [37] and ASTM D7405 [38]), the percentage error values among the test results of the RTFO (equivalent time) and PAV tests were compared. The ASTM D7175 standard [37] states that the acceptance range for G*/sinδ (RTFO residue) is 9.0%, and for G*.sinδ (PAV Residue), it is 13.8%. For the J_nr_ parameter, the ASTM D7405 standard [38] states that the maximum difference is 38.3% at a stress level of 0.1 kPa and 26.6% at a stress level of 3.2 kPa. The LAS standard (DNIT 439 [39]) does not present an acceptable range for the two test results. The percentage error differences between the RTFO and PAV tests (Table 5) were lower than the acceptable values stated in the standards.

The equivalent time proposed for the RTFO test for long-term ageing presented promising results in terms of rheological parameters compared to the PAV results. For the oscillatory tests in the DSR, specifically for the G*.sinδ parameter, the obtained results were too close. For the MSCR and LAS parameters, the difference was higher, and these higher values can be attributed to the deformation regimen proposed by the test, where the viscoelastoplastic regimen is tested, so higher variability is expected. On the other hand, for the oscillatory test, the test procedure is performed under the viscoelastic regimen.

A straightforward methodology was used to simulate long-term ageing using the RTFO, focusing on developing countries, as time test duration and equipment costs (mainly for the PAV) can represent a limiting factor to perform the tests. Replacing the PAV test is not recommended, but RFTO provides an alternative. It has been suggested that when using RTFO cycles to simulate long-term ageing, at least one sample should be aged using the PAV for comparison.

This work has limitations since the tests were conducted only in a laboratory, and a field comparison is essential, especially to determine the influence of mixture production, mixture type, the location of the implanted pavement, and the position in the pavement surface layer. Also, only unmodified Brazilian asphalt PEN 50/70 was tested, and the methodology applied should be extended for other asphalt types from different sources. Future research must incorporate chemical characteristic evaluations using Fourier-transform infrared spectroscopy (FTIR) and aim to determine the influence of UV radiation on the ageing process.

## 4. Conclusions

The main objective of this work was to establish the equivalent time that the RTFO takes to simulate long-term ageing compared to the PAV. The Brazilian unmodified asphalt PEN 50/70 (PG 58-XX) was used to perform the tests.

The asphalt samples were aged in a standard RTFO test at different cycle times (85, 170, 255, and 340 min). Then, the samples were subjected to rheological tests such as MSCR and LAS. Also, the rutting and fatigue SUPERPAVE parameters were measured. The same tests were conducted on PAV samples.

Based on the rheological parameter results for the RFTO cycles and PAV, the equivalent time was estimated via linear regression. The following conclusions were drawn.

The more the asphalt is aged the more its stiffness increases. However, as both the RTFO cycles and temperature increased, the stiffness increase occurred on a minor percentual scale. Phase angle was not a suitable indicator parameter for ageing evaluation. It was also observed that the RTFO ageing sequential times influenced the material stiffness, which increased, as noted by the J_nr_ reduction.

The test results indicated that the equivalence between RTFO and PAV, in terms of time, was between 255 min and 340 min, corroborating the results of previous studies [28]. This time range was observed for the SUPERPAVE rutting (G*/sinδ) and fatigue (G*.sinδ) parameters, the J_nr_ parameter from the MSCR test at 58 °C, and the LAS N_f_ parameter (19 °C, according to the Brazilian standard).

Through using linear regression and the PAV results as a reference, the equivalent time was determined to be 300 min. The rheological test results for the asphalt samples aged in the RTFO for 300 min were compared to those obtained via long-term ageing using the PAV. The acceptable difference range between the two test results, considering the acceptable values outlined by the relevant test standards, was evaluated. Our results proved that the differences between the test results for the RTFO samples aged at 300 min and that of the PAV samples did not exceed the limits, indicating that the equivalent time obtained is suitable.

## Figures and Tables

**Table 1:** Asphalt PEN 50/70 characterization test results and limits of the Brazilian specification.

Test	Unit	Results	Limits ^3^	Standard
Penetration (25 °C, 5 s, 100 g)	0.01 mm	52	50–70	[33]
Softening point ^1^	°C	48	46 (min.)	[34]
Apparent viscosity ^2^ (135 °C)	cP	286	274 (min.)	[35]
Ductility (25 °C)	cm	100	60 (min.)	[36]
RTFO (163 °C, 85 min)				[6]
Mass loss	%	0.11	0.5 (max.)	[6]
Change in softening point	^o^C	3.3	8 (max.)	[34]
Retained penetration	%	66	55 (min.)	[33]

^1^ Ring and ball method; ^2^ Brookfield viscometer, spindle 21, 20 rpm; ^3^ [26].

**Table 2:** Short-term ageing test times.

Nomenclature	Times	Duration (Min)
PEN1 ^1^	1 × RTFO	85
PEN2	2 × RTFO	170
PEN3	3 × RTFO	255
PEN4	4 × RTFO	340

^1^ Reference.

**Table 3:** Results of the parameters for high-temperature PG.

Parameters	Temperature (°C)		
	58	64	70
Original asphalt			
G* (kPa)	2.06	0.96	0.47
δ (^o^)	87.8	88.4	88.3
G*/sinδ (kPa)	2.06	0.96	0.47
After RTFO			
G* (kPa)	3.713	1.62	0.74
δ (^o^)	86.3	87.5	88.2
G*/sinδ (kPa)	3.72	1.62	0.74

**Table 4:** Rheological parameters at high temperatures.

Asphalt/Nomenclature	RTFO Times (Min)	T (°C)	G (kPa)	δ (°)
PEN 50/70 ^1^	0	58	2.06	87.8
		64	0.96	88.4
		70	0.47	88.3
PEN1	85	58	3.72	86.3
		64	1.62	87.4
		70	0.74	88.2
PEN2	170	58	6.70	84.0
		64	2.88	85.7
		70	1.29	87.1
PEN3	255	58	9.14	82.4
		64	3.86	84.3
		70	1.71	87.0
PEN4	340	58	13.83	79.5
		64	6.07	81.9
		70	2.79	83.9

^1^ Reference.

**Table 5:** Rheological parameters at intermediate temperatures.

Asphalt/Nomenclature	RTFO Times (Min)	T (°C)	G (kPa)	δ (°)
PEN2	170	19	8.12	48.5
		22	4.96	53.3
		25	2.85	58.2
PEN3	255	19	8.25	46.2
		22	5.16	50.8
		25	3.08	55.4
PEN4	340	19	11.84	43.6
		22	7.62	47.9
		25	4.61	52.3

**Table 6:** Equivalent time evaluated for each test parameter.

Test Parameters	PAV	Equivalent Time RTFO (Min)
G*/sinδ (kPa) @ 58 °C	11.90	301
G*/sinδ (kPa) @ 64 °C	4.93	295
G*.sinδ (kPa) @ 19 °C	10.20	291
G*.sinδ (kPa) @ 22 °C	6.50	293
G*.sinδ (kPa) @ 25 °C	3.92	292
J_nr0.1_ (kPa^−1^) @ 58 °C	0.072	309
J_nr3.2_ (kPa^−1^) @ 58 °C	0.077	302
N_f_ (cycles), 1.25% @ 19 °C	571,776	316
N_f_ (cycles), 2.50% @ 19 °C	40,682	303
Mean (min)		300
Standard deviation (min)		8.4

**Table 7:** Comparison of rheological parameters obtained in the RTFO (equivalent time) and PAV tests.

Test Parameters	PAV	PENeq	Difference (%)
G*/sinδ (kPa) @ 58 °C	11.9	12.6	−5.7
G*.sinδ (kPa) @ 19 °C	10.2	10.1	0.4
G*.sinδ (kPa) @ 22 °C	6.5	6.4	1.5
G*.sinδ (kPa) @ 25 °C	3.9	3.8	2.5
J_nr0.1_ (kPa^−1^) @ 58 °C	0.07	0.065	9.7
J_nr3.2_ (kPa^−1^) @ 58 °C	0.08	0.068	11.7
N_f_ (cycles), 1.25% @ 19 °C	571,776	519,216	9.2
N_f_ (cycles), 2.50% @ 19 °C	40,682	37,521	7.8

## Data Availability

All data are shown in the manuscript.
